# RNA Binding by Histone Methyltransferases Set1 and Set2

**DOI:** 10.1128/MCB.00165-17

**Published:** 2017-06-29

**Authors:** Camille Sayou, Gonzalo Millán-Zambrano, Helena Santos-Rosa, Elisabeth Petfalski, Samuel Robson, Jonathan Houseley, Tony Kouzarides, David Tollervey

**Affiliations:** a Wellcome Trust Centre for Cell Biology, University of Edinburgh, Edinburgh, Scotland; b Gurdon Institute and Department of Pathology, University of Cambridge, Cambridge, United Kingdom; c Epigenetics Programme, The Babraham Institute, Cambridge, United Kingdom

**Keywords:** Set1, Set2, RNA, chromatin, transcription, yeast, UV cross-linking, RNA-protein interaction, histone methyltransferase, histone modification

## Abstract

Histone methylation at H3K4 and H3K36 is commonly associated with genes actively transcribed by RNA polymerase II (RNAPII) and is catalyzed by Saccharomyces cerevisiae Set1 and Set2, respectively. Here we report that both methyltransferases can be UV cross-linked to RNA *in vivo*. High-throughput sequencing of the bound RNAs revealed strong Set1 enrichment near the transcription start site, whereas Set2 was distributed along pre-mRNAs. A subset of transcripts showed notably high enrichment for Set1 or Set2 binding relative to RNAPII, suggesting functional posttranscriptional interactions. In particular, Set1 was strongly bound to the *SET1* mRNA, Ty1 retrotransposons, and noncoding RNAs from the ribosomal DNA (rDNA) intergenic spacers, consistent with its previously reported silencing roles. Set1 lacking RNA recognition motif 2 (RRM2) showed reduced *in vivo* cross-linking to RNA and reduced chromatin occupancy. In addition, levels of H3K4 trimethylation were decreased, whereas levels of dimethylation were increased. We conclude that RNA binding by Set1 contributes to both chromatin association and methyltransferase activity.

## INTRODUCTION

A major function of chromatin in eukaryotic cells is the regulation of gene expression in the form of RNA transcripts. It therefore seemed likely that there would be an extensive interplay between the transcriptome and chromatin-associated factors (1). Consistent with this idea, chromatin proteins were identified by mass spectrometry following UV cross-linking and purification of RNA-protein complexes in both yeast and human cells (2–4). Moreover, recent analyses of a panel of chromatin-associated proteins identified 24 protein-RNA interactions that could be recovered through formaldehyde-based cross-linking in human cells (5).

Two prominent modifications in chromatin are the methylation of histone H3 at lysine 4 (H3K4) and lysine 36 (H3K36). In the budding yeast Saccharomyces cerevisiae, these modifications are catalyzed by the Set1 and Set2 methyltransferases, respectively. During transcription, the large catalytic subunit of RNA polymerase II (RNAPII), Rpo21 in yeast, undergoes dynamic phosphorylation/dephosphorylation events within the heptad repeats forming the carboxy-terminal domain (CTD). These events help coordinate the recruitment of transcription and RNA processing factors to the elongating RNAPII and nascent transcript (reviewed in references 5–7). Set1 functions within the complex of proteins associated with Set1 (COMPASS or Set1C) (reviewed in references 8 and 9), which is brought to RNAPII through interactions with the PAF complex when the CTD is phosphorylated on serine 5 (RNAPII-S5P). The recruitment of Set2 to the elongating RNAPII occurs when serines 2 and 5 are phosphorylated and also requires the PAF complex (reviewed in references 10–12).

Trimethylation of H3K4 (H3K4me3) is a characteristic feature of the 5′ regions of actively transcribed genes, and this correlation has often led to an expectation that Set1 functions to stimulate transcription. However, from the earliest analyses of yeast, Set1 was implicated in gene silencing (13, 14). Subsequent analyses implicated Set1 in the repression of many genes (15, 16), with more obvious effects under stress conditions (17). In addition, Set1 has been implicated in the transcriptional silencing of retrotransposons in S. cerevisiae (18, 19) and in Schizosaccharomyces pombe (20–22). Set1-dependent silencing of Ty1 retrotransposons is mediated by a noncoding, antisense transcript (19). Set1 is also implicated in the silencing of RNAPII transcription from the intergenic spacer (IGS) regions located between the ribosomal DNA (rDNA) genes (13, 23). H3K36 methylation is found throughout protein-coding genes and prevents the initiation of transcription at cryptic sites via the recruitment of the Rpd3S histone deacetylase complex (24–26). H3K36 methylation has also been reported to regulate pre-mRNA splicing (27).

Yeast Set1 has two putative RNA recognition motifs (RRMs) that are implicated in Set1 function, suggesting that it might bind RNA *in vivo* (28, 29). Set2 does not harbor an evident RNA-binding motif but was identified in systematic analyses of yeast RNA-interacting proteins (2, 30). However, *in vivo* targets for these potential RNA-binding activities have not been reported.

To identify potential direct RNA interactions for Set1 and Set2, we employed UV cross-linking and analysis of cDNAs (CRAC). This analysis showed that both Set1 and Set2 associate with almost all RNAPII transcripts. However, the binding of Set1 and Set2 relative to transcription rates is variable. Transcripts showing high relative binding by Set1 and Set2 are candidates for posttranscriptional regulation. Our results show that Set1 interactions with RNA are mediated predominately by RRM2 and indicate that contacts with RNA reinforce both chromatin binding and methyltransferase activity.

## RESULTS

### Set1 and Set2 bind to RNA *in vivo*.

**FIG 1:**
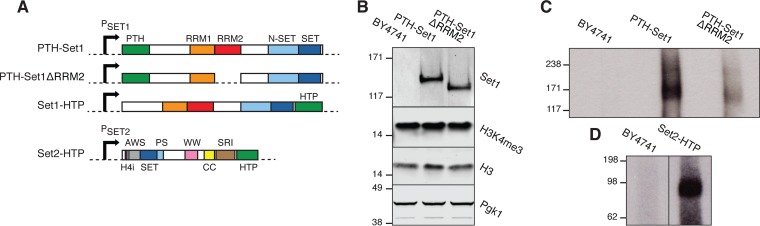
Set1 and Set2 interact with RNA *in vivo*. (A) Domain organization of fusion proteins used in this study. RRM, RNA recognition motif; H4i, H4-interacting domain; AWS, associated with SET; PS, post-SET; WW, tryptophan-rich domain; CC, coiled-coil domain; SRI, Set2-Rpb1 interaction domain; PTH, protein A-TEV-His_6_ tag; HTP, His_6_-TEV-protein A tag. P_SET1_ and P_SET2_ are *SET1* and *SET2* promoters, respectively. (B) Western blots showing the protein abundance in the samples used in panel C. Cells were grown in minimal medium lacking tryptophan and UV cross-linked. The input lysate was analyzed with antibodies against H3K4me3, H3, and Pgk1 (loading controls). Molecules eluted from IgG beads using TEV protease were analyzed with anti-Set1 antibodies. (C and D) SDS-PAGE and autoradiography of the 5′-^32^P-labeled, cross-linked RNAs after purification of the tagged proteins or after mock purification of proteins from the untagged strain (BY4741).

To perform CRAC, the endogenous *SET1* gene was tagged with either an N-terminal protein A-tobacco etch virus (TEV) protease cleavage site-His_6_ (PTH) tag or a C-terminal His_6_-TEV-protein A (HTP) tag. The endogenous *SET2* gene was tagged with C-terminal HTP. All constructs were expressed under the control of the endogenous promoter and were the sole form of Set1 or Set2 in the cell (Fig. 1A). In strains expressing only PTH-Set1 or Set2-HTP, global H3K4me3 and H3K36 methylation levels and cell growth were similar to those of the wild type (WT) (Fig. 1B; see also Fig. S1A to C in the supplemental material). In contrast, Set1-HTP strains lacked detectable H3K4me3, consistent with data from previous reports of C-terminally tagged Set1 proteins (20, 28, 31) (Fig. S1A), and were more slow growing than the wild-type strain (Fig. S1C). However, the protein level was unaffected (Fig. S1A), in contrast to a previous report that a loss of methyltransferase activity results in protein depletion (32). This discrepancy likely reflects structural differences in the alleles used.

To test for *in vivo* RNA binding, actively growing cells were UV irradiated, the tagged proteins were purified, and cross-linked RNAs were labeled and visualized by SDS-PAGE and autoradiography. This analysis showed that PTH-Set1, Set1-HTP, and Set2-HTP were all bound to RNAs *in vivo* (Fig. 1C and D and Fig. S1D).

Set1 RRM2 was predicted to be a functional RNA-binding domain, whereas RRM1 appeared less likely to interact with RNA, and this was supported by data from previous *in vitro* assays (28). Moreover, a deletion overlapping RRM1 reduced Set1 levels, whereas a construct lacking only RRM2 was stable (32). To assess RNA binding by Set1, we therefore deleted RRM2 (residues 415 to 494) from the PTH-Set1 strain to obtain PTH-Set1ΔRRM2 (Fig. 1A). The abundance of PTH-Set1ΔRRM2 was similar to that of PTH-Set1, and the deletion did not clearly alter global H3K4me3 levels (Fig. 1B) or growth (Fig. S1C). In CRAC analyses, PTH-Set1ΔRRM2 greatly reduced, but did not abolish, RNA cross-linking relative to PTH-Set1 (Fig. 1C). We therefore conclude that most of the RNA-binding activity in Set1 is attributable to RRM2. Residual binding observed in PTH-Set1ΔRRM2 may result from RRM1.

### Set1 and Set2 associate with nascent RNAPII transcripts.

RNA fragments purified with Set1 and Set2 (from strains PTH-Set1, PTH-Set1ΔRRM2, Set1-HTP, and Set2-HTP) were converted to cDNA and sequenced. RNA was also recovered following mock purification from the untagged strain (BY4741) and represents the background of the experiment. At least 3 replicate data sets each were obtained for PTH-Set1, PTH-Set1ΔRRM2, Set1-HTP, and Set2-HTP (see Table S1 in the supplemental material).

To better estimate the relative *in vivo* binding of PTH-Set1 and PTH-Set1ΔRRM2 to RNA, cross-linked and barcoded samples were mixed prior to SDS-PAGE separation and reverse transcription-PCR (RT-PCR) amplification. Following demultiplexing, the number of reads recovered for PTH-Set1ΔRRM2 was about 3-fold lower than that for PTH-Set1 (Fig. S1E), consistent with the reduced binding observed from the autoradiography gels (Fig. 1C). Substantially fewer reads were recovered for the BY4741 untagged control.

**FIG 2:**
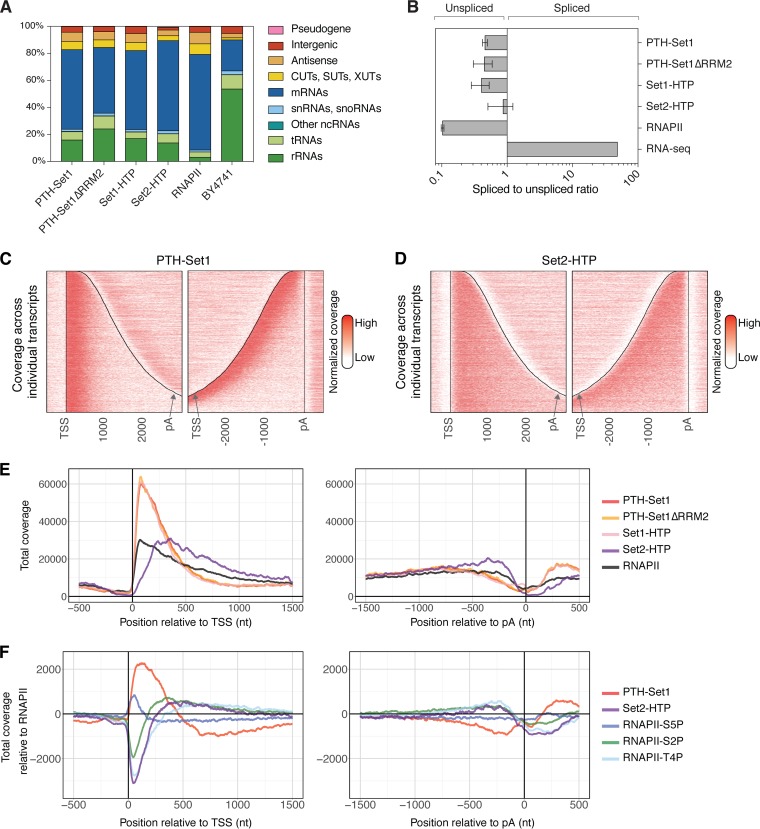
Set1 is enriched near the TSS, while Set2 binds across nascent RNAPII transcripts. (A) Distribution of reads across transcript classes in the CRAC data sets. Replicates have been averaged. Rpo21-HTP represents RNAPII. (B) Relative recovery of spliced mRNAs versus unspliced pre-mRNAs, expressed as a ratio of RNA fragments spanning exon-exon junctions to those spanning intron-exon and exon-intron junctions. Error bars represent standard deviations from the replicates listed in Table S1 in the supplemental material. (C and D) Distribution of PTH-Set1 (C) and Set2-HTP (D) across individual mRNAs in reads per million of RNAPII transcripts. Transcripts are aligned to the TSS (left) and the pA site (right), respectively. Distances are indicated in nucleotides. The corresponding total coverages are shown in panel E. (E) Metagene analysis of PTH-Set1, PTH-Set1ΔRRM2, Set1-HTP, Set2-HTP, and RNAPII (Rpo21-HTP) across mRNAs, in reads per million of RNAPII transcripts. Transcripts are aligned to the TSS (left) or the pA site (right). (F) Metagene analysis of PTH-Set1, RNAPII-S5P, Set2-HTP, RNAPII-S2P, and RNAPII-T4P enrichment relative to total RNAPII, across mRNAs aligned to their TSS (left) or pA site (right). Relative enrichment was calculated as log_2_(protein coverage/total Rpo21-HTP coverage). The enrichment across individual mRNAs is shown in Fig. S3E and S3F for PTH-Set1 and Set2-HTP.

The distribution of reads among RNA classes showed that both Set1 and Set2 predominately bound mRNAs (Fig. 2A). Compared to BY4741, Set1, but not Set2, was also enriched for binding to other non-protein-coding RNAs (ncRNAs) transcribed by RNAPII, including SUTs, CUTs, XUTs, and intergenic transcripts. For comparison, we also show the distribution of the catalytic subunit of RNAPII (Rpo21-HTP) using previously reported CRAC data (33). PTH-Set1, PTH-Set1ΔRRM2, and Set1-HTP showed broadly similar distributions (Fig. 2A). PTH-Set1ΔRRM2 samples showed more rRNA and tRNA reads than PTH-Set1 (Fig. 2A), although this is likely to reflect a higher background level due to reduced RNA binding rather than a difference in endogenous RNA target classes. This indicated that the loss of RRM2 greatly reduces the affinity for RNA (Fig. 1C) but has a limited impact on specificity (Fig. 2A).

Pre-mRNA splicing is largely cotranscriptional in yeast (34–36). Therefore, the presence of unspliced RNAs in CRAC data sets generally reflects protein interactions with nascent transcripts. To assess whether Set1 and Set2 bind cotranscriptionally, the recovery of spliced and unspliced transcripts from intron-containing genes was calculated as reported previously (33, 37). The ratio of reads spanning exon-exon junctions (spliced) relative to exon-intron plus intron-exon junctions (unspliced) was below 1 for both Set1 and Set2 (Fig. 2B), indicating predominant binding to unspliced, nascent pre-mRNAs. For Set2, the ratio was higher than that for Set1, consistent with Set2 binding later during transcription. PTH-Set1, PTH-Set1ΔRRM2, and Set1-HTP showed similar ratios. Since both Set1 and Set2 bound a higher proportion of spliced transcripts than RNAPII, we addressed their possible posttranscriptional association with mRNAs by comparing their binding to mRNA stability, determined by transcriptome sequencing (RNA-seq) following RNAPII inhibition (38). Enrichment of Set1 and Set2 relative to RNAPII did not increase with the mRNA half-life (Fig. S2A to D), strongly indicating that Set1 and Set2 are not predominantly bound to mature mRNAs. We conclude that Set1 and Set2 are directly associated with nascent RNAPII transcripts, consistent with their function during transcription.

### Set1 binding is enriched near the TSS, while Set2 binds across transcripts.

Binding profiles on mRNAs for PTH-Set1 and Set2-HTP were aligned via the transcription start site (TSS) or the poly(A) (pA) site (Fig. 2C to E). This showed that PTH-Set1 binding was strongly enriched over the 5′ end of mRNAs, from the TSS to nucleotide (nt) +500. In contrast, Set2-HTP binding was more distributed along transcripts, from nt +150 after the TSS to nt −150 before pA sites. This pattern was also clearly visible on individual mRNAs (see Fig. S3A to D in the supplemental material). We also compared PTH-Set1 binding with the residual binding of PTH-Set1ΔRRM2 and with binding of Set1-HTP. All three proteins showed similar profiles, suggesting that, once Set1 was bound to RNA, RRM2 did not significantly influence its distribution along mRNAs (Fig. 2E) and that the lack of methylation activity also did not influence the Set1 distribution across mRNAs (Fig. 2E).

The RNAPII distribution across transcripts shows higher density over the TSS-proximal region (Fig. 2E), likely reflecting a substantial level of premature transcription termination in the 5′-proximal region (33, 37, 39–42). To account for this uneven transcript distribution, binding of Set1 and Set2 was expressed relative to RNAPII coverage. Relative coverage was calculated as the log_2_(protein coverage/Rpo21-HTP coverage) and plotted along mRNAs (33). Set1 was strongly enriched relative to RNAPII at the 5′ ends of mRNAs (Fig. 2F and Fig. S3E). Set2 was relatively depleted from the promoter-proximal region, and its level progressively rose to peak at around nt +400 from the TSS and then remained elevated along transcripts (Fig. 2F and S3F). These profiles are consistent with previously reported distributions of H3K4me3 and H3K36me3 on chromatin (43).

Set1 was reported to be recruited to chromatin when the CTD is phosphorylated on serine 5, whereas Set2 is recruited when both serine 5 and serine 2 are phosphorylated (44). We therefore compared the relative distributions of Set1 and Set2 to RNAPII with the five types of CTD phosphorylation states (Y1P, S2P, T4P, S5P, and S7P), which were recently mapped to RNA by using a CRAC-related technique (33). Both Set1 and RNAPII-S5P peaked close to the TSS, but their distributions differed significantly. RNAPII-S5P was strongly enriched across the first 130 nt from the TSS and was then sharply depleted. In contrast, the enrichment profile of Set1 extended further in the 3′ direction (Fig. 2F). The observation of high levels of Set1 binding over regions with low S5P levels strongly indicates that this is not the only determinant of the Set1 distribution over RNAs. The Set2 profile closely resembled those of both RNAPII-S2P and RNAPII-T4P (Fig. 2F), consistent with Set2 recruitment to the CTD being modified with S2P and perhaps T4P.

Set1 and, to a lesser extent, Set2 were bound to ncRNAs, including SUTs, CUTs, XUTs, and intergenic and antisense transcripts, in addition to mRNAs (Fig. 2A). To compare Set1 and Set2 enrichment profiles over mRNAs and ncRNAs, we used an expression-matched subset of mRNAs, SUTs and CUTs, based on their total RNAPII CRAC signal over the first 300 nt (33). Set1 and Set2 were less enriched on SUTs than on mRNAs and even less enriched on CUTs (Fig. S3G). On the same sets of transcripts, RNAPII-S5P profiles were similar, whereas RNAPII-S2P showed decreased enrichment, as previously reported (33). We speculate that the ncRNAs, particularly CUTs, undergo very rapid degradation that occurs immediately following transcription and may even be partially cotranscriptional, greatly restricting the time available for Set1 or Set2 associations.

The Set1 and Set2 RNA-binding profiles clearly support predominately cotranscriptional recruitment. However, the correlation between Set1 and RNAPII-S5P indicates that this is not the sole key recruitment factor, while data from the ncRNA analyses suggest that the RNA association is at least transiently retained with the released transcripts.

### High-level binding of Set1 and Set2 to specific transcripts suggests functional interactions.

**FIG 3:**
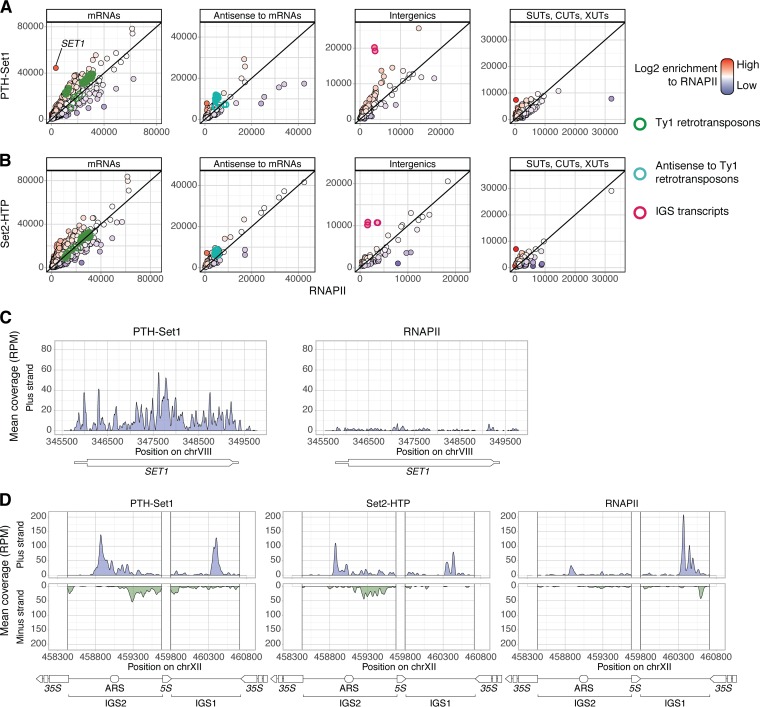
Some transcripts show high enrichment for Set1 or Set2 relative to RNAPII. (A and B) PTH-Set1 (A) or Set2-HTP (B) coverage over genomic features (mRNAs, transcripts antisense to mRNAs, intergenic transcripts, SUTs, CUTs, and XUTs) is plotted against RNAPII coverage (Rpo21-HTP). The fill color of the points represents the enrichment for Set1 or Set2 relative to RNAPII. Some classes of transcripts are highlighted, as indicated on the right. Other RNA classes are shown in Fig. S5C in the supplemental material. (C and D) Coverage, in reads per million (RPM) of RNAPII transcripts, at loci where Set1 is enriched over RNAPII. (C) *SET1* locus. The transcription unit is represented under the plots, with the thicker box corresponding to the coding sequence. (D) A rDNA intergenic spacer (IGS) region. rRNA genes appear white on the plots.

We hypothesized that transcripts with functionally relevant Set1 or Set2 binding would show higher enrichment than with RNAPII (i.e., transcription rate). The coverage of Set1 or Set2 over genomic features, including mRNAs and noncoding transcripts, was plotted against RNAPII coverage, as determined by cross-linking of Rpo21-HTP (Fig. 3A and B). Overall, Set1 and Set2 binding was broadly correlated with RNAPII coverage. There was, however, some heterogeneity, with a subset of transcripts showing high-level binding despite low levels of transcription.

Set1 showed high relative binding to *SET1* mRNA (Fig. 3A and C). PTH-Set1 binding along the mRNA was broadly distributed and, in contrast to most mRNAs, did not show a clear 5′ peak (Fig. 3C), indicating that the interaction is at least not only cotranscriptional. The N-terminal tag is present on the nascent peptide throughout translation, whereas the C-terminal tag is synthesized just before dissociation from the ribosome, and binding to *SET1* mRNA was strongly reduced for Set1-HTP compared to PTH-Set1 (see Fig. S4A in the supplemental material). These observations are consistent with the previously reported cotranslational binding of *SET1* mRNA by Set1 and three other COMPASS components (45). Differences in the recovery of *SET1* mRNA between different Set1 strains did not result from altered mRNA abundance (Fig. S4C).

Set1 was also enriched on a group of partially overlapping, ncRNA transcripts derived from the rDNA intergenic spacer regions (IGS ncRNAs) and over Ty1 retrotransposons, with strong binding to both mRNAs and antisense transcripts (Fig. 3A and D and S4B). RT-quantitative PCR (qPCR) analyses showed that these transcripts are unaltered in the PTH-Set1, PTH-Set1ΔRRM2, and Set2-HTP strains, relative to Rpo21-HTP or wild-type strain BY4741 (Fig. S4D and E). In contrast, Set1-HTP showed increased transcript levels, notably for the rDNA IGS, suggesting that the Set1 histone methyltransferase activity, but not RNA binding, may be involved in regulating the abundance of these ncRNAs. PTH-Set1ΔRRM2 and PTH-Set1 showed similar enrichments of RNAPII over the different sequence features (Fig. S5A and B). We conclude that while RRM2 strongly contributes to the level of RNA association, it is not primarily responsible for the specificity of RNA binding by Set1.

Comparison of Set2 to RNAPII identified only a few mRNAs with high relative Set2 binding (Fig. 3B). However, Set2 was enriched over the rDNA IGS ncRNAs (Fig. 3B and D) and, most clearly, over a subset of the box C/D class of small nucleolar RNAs (snoRNAs) (Fig. S5C). The PAF complex and, less clearly, Set2 were previously implicated in snoRNA 3′-end formation (46, 47), suggesting a possible link between this process and Set2 RNA binding.

To check whether the RNA-binding activity regulates transcript abundance, genes showing differential expression were identified in strains carrying *set1*Δ (18) or *set2*Δ (see Materials and Methods). However, for both proteins, the differentially expressed genes corresponded to transcripts with low coverage for PTH-Set1, Set2-HTP, and RNAPII, indicating their low expression levels. No clear enrichment was seen for mRNAs showing low- or high-level binding of Set1 and Set2 relative to RNAPII (Fig. S5D).

In conclusion, despite cotranscriptional binding to all RNAPII transcription units, Set1 and Set2 were strongly enriched on small numbers of transcripts. For Set1, these transcripts largely represent known silencing targets.

### RNA binding stabilizes interactions of Set1 with chromatin and regulates the balance between H3K4 di- and trimethylation.

**FIG 4:**
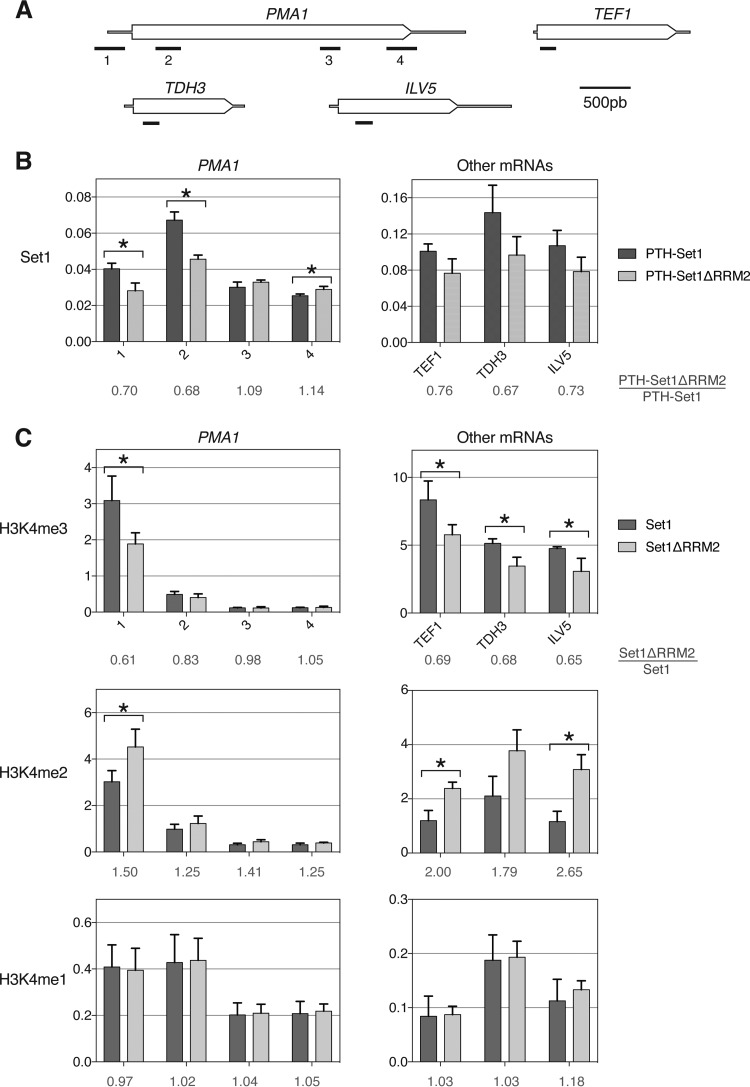
RNA binding stabilizes interactions of Set1 with chromatin. (A) Schematic representation of the genes analyzed. The transcription unit is represented, with the coding sequences being thicker. Bars indicate PCR products. (B) Set1 ChIP in the PTH-Set1 and PTH-Set1ΔRRM2 strains. Associated DNA was analyzed by qPCR, and the signal is expressed as a percentage of the input DNA. Error bars represent the standard deviations from biological triplicates. * indicates a different signal with a *P* value of <0.05, calculated with Student's *t* test. (C) H3K4me3, H3K4me2, and H3K4me1 ChIP in the wild-type and Set1ΔRRM2 strains. The signal is normalized to the total H3 signal. Error bars represent the standard deviations from biological triplicates. * indicates a different signal with a *P* value of <0.05, calculated with Student's *t* test.

The potential contribution of RNA binding to stabilizing the association of Set1 with chromatin *in vivo* was assessed by chromatin immunoprecipitation (ChIP) followed by qPCR. The distribution of Set1 along *PMA1* matched data from previous reports (32, 48), with stronger cross-linking near the 5′ end. Binding of PTH-Set1ΔRRM2 to chromatin was ∼30% reduced compared to that of PTH-Set1 at the 5′ end (primer pairs 1 and 2) (Fig. 4A and B), where Set1 RNA binding peaked (Fig. 2C and E; see also Fig. S3A to D in the supplemental material). Binding to the 3′ end of *PMA1* (primer pairs 3 and 4) was similar for both proteins (Fig. 4A and B). Reductions in the binding of PTH-Set1ΔRRM2 of ∼25 to 30% were seen for three other genes tested (*TEF1*, *TDH3*, and *ILV5*) (Fig. 4A and B). The data indicate that reduced RNA binding by Set1 leads to weaker interactions with chromatin, but this may be specific for 5′ regions that show high Set1-RNA interactions.

We then tested whether the reduced chromatin occupancy caused by the RRM2 deletion affected H3K4 methylation. ChIP-qPCR was performed to assess the levels of H3K4me1, H3K4me2, and H3K4me3 in the strain expressing Set1ΔRRM2 compared to the wild-type strain expressing native Set1. The level of H3K4me3 was reduced by 20 to 30% in the Set1ΔRRM2 strain in the 5′ regions of *PMA1*, *TEF1*, *TDH3*, and *ILV5*, whereas H3K4me3 was unchanged near the 3′ end of *PMA1* (Fig. 4C). In contrast, we observed similarly increased levels of H3K4me2 at all loci tested. In the case of *PMA1*, this increase was more pronounced at the 5′ end (Fig. 4C). We observed no change in H3K4me1 (Fig. 4C). A significant global change in the three methylation states could not be detected by Western blotting, likely due to the lack of sensitivity of the method compared to ChIP (Fig. S6A and S6B). This shows that RRM2 is required for the normal balance between H3K4me3 and K3K4me2, particularly at the 5′ ends of genes.

These results demonstrate that RRM2 is functionally important for Set1 targeting at chromatin and for the methylation of H3K4. We propose that RNA binding participates in Set1 recruitment and/or stabilization at chromatin, thereby contributing to H3K4 methylation patterns.

## DISCUSSION

This study presents high-resolution, strand-specific, transcriptome-wide mapping of two major histone methyltransferases, Set1 and Set2, which are conserved from yeast to human. Both proteins directly interacted with RNA *in vivo* (Fig. 1C and D) and showed preferential interactions with nascent RNAPII transcripts (Fig. 2A and B; see also Fig. S2 in the supplemental material), consistent with their association with transcribing RNAPII. Set1 was enriched at the 5′ end of mRNAs, whereas Set2 was distributed along transcripts (Fig. 2C to F and Fig. S3A to F), matching the distributions of H3K4me3 and H3K36me3 on chromatin, respectively (43).

Binding of Set1 and Set2 was detected for all active RNAPII transcription units. However, some RNAs showed high protein binding relative to their transcription rate, particularly for Set1 (Fig. 3A and B and Fig. S5C), suggesting posttranscriptional interactions. *SET1* mRNA was one of the most enriched transcripts for Set1 binding. The broad distribution of Set1 and the lack of 5′ bias along *SET1* mRNA indicate posttranscriptional binding (Fig. 3C). This interaction was previously proposed to be cotranslational (45), and the reduction in Set1 mRNA binding observed for Set1-HTP for but not PTH-Set1ΔRRM2 would be consistent with this conclusion (Fig. S4A).

Ty1 mRNAs and Ty1 antisense transcripts were found to be strongly enriched for Set1 binding. IGS ncRNAs from the rDNA repeats were enriched for both Set1 and Set2 (Fig. 3A, B, and D and Fig. S4B). Strikingly, Set1 was previously shown to participate in the silencing of retrotransposons (18, 19) and IGS regions (13, 23), supporting the model that functionally important Set1 targets would show preferential binding relative to RNAPII.

Phosphorylation and dephosphorylation of the CTD of the large subunit of RNAPII coordinate the recruitment of numerous factors, including Set1 and Set2 (6, 8). The distribution of Set2 along genes was similar to that of RNAPII-S2P, consistent with its reported role in recruitment. However, Set2 also closely matched RNAPII-T4P (Fig. 2F), suggesting the possible involvement of this CTD modification in Set2 recruitment. Surprisingly, the distribution of Set1 was distinct from that of RNAPII-S5P (Fig. 2F), strongly indicating that additional parameters help define the Set1 localization along transcripts.

The Set1ΔRRM2 protein showed reduced chromatin association in ChIP analyses (Fig. 4A and B), indicating that RNA binding functions in the recruitment of Set1 to chromatin and/or stabilizes the association. Consistent with this conclusion, it was previously shown that a truncated version of Set1 containing only the SET domain and most of the N-SET had reduced chromatin occupancy in yeast (32). Notably, analyses at different sites along the *PMA1* gene revealed clear differences in chromatin occupancy only in the 5′ region. This suggests a potential correlation between the stabilization of chromatin association and high-level RNA binding. Consistent with this model, the absence of RRM2 also led to reduced H3K4me3 and increased H3K4me2 levels, at the 5′ ends of genes (Fig. 4C), demonstrating that RRM2 is required for the correct distribution of H3K4me3 and H3K4me2.

*In vivo* and *in vitro* experiments previously showed that the pattern of mono-, di-, and trimethylation deposited by Set1 correlated with the interaction time of the COMPASS complex with its nucleosome substrate, with monomethylation occurring virtually immediately, followed by dimethylation and, finally, trimethylation (49). Other parameters, such as the COMPASS complex subunit composition, also direct the distribution of the three methylation states (9, 50). We propose that RNA binding of Set1 via RRM2 near the TSS stabilizes the association of Set1/COMPASS with chromatin, promoting the formation of H3K4me3 at the 5′ ends of genes. Due to reduced RNA binding, the Set1ΔRRM2-chromatin interaction is weaker or more transient, leading to higher levels of H3K4me2. A major role of H3K4me2 is the recruitment of the Set3 histone deacetylase complex, which deacetylates histones in 5′ regions of transcription units and participates in H3K4me2 maintenance (51). This helps regulate overlapping noncoding transcription and contributes to epigenetic transcriptional memory (50, 52). We speculate that the disruption of RNA binding by Set1 adversely affects these processes.

The results reported here contribute to the understanding of the cross talk between RNA synthesis and the modulation of chromatin structure. Recent studies have identified a large number of RNA-interacting proteins in eukaryotic cells. Given the key role of chromatin in the regulation of RNA synthesis, it might be anticipated that functional RNA interactions will be particularly prevalent among the readers, writers, and erasers of epigenetic chromatin marks. However, previous analyses reported comparatively fewer examples of such interactions. In this context, the identification of RNA-binding activity by the two major histone methyltransferases in yeast is perhaps not entirely unexpected. Many analyses revealed substantial functional redundancy among epigenetic regulatory systems in yeast. We anticipate that the importance of RNA interactions by Set1 and Set2 will be more evident in cells that are also deficient in other epigenetic pathways or are undergoing rapid changes in the gene expression program, which will be frequent for yeast growing in the natural environment.

## MATERIALS AND METHODS

### Strains.

Yeast analyses were performed with strains derived from BY4741, except for the RNA-seq experiment, which was done in the W303 background. All strains used are listed in Table S2 in the supplemental material. Oligonucleotides are listed in Table S3 in the supplemental material. The PTH-Set1 strain was obtained by integrating a sequence encoding a PTH (2× protein A-TEV-His) tag at the 5′ end of *SET1*, resulting in the expression of an N-terminally tagged protein expressed from the endogenous *SET1* promoter. The generation of this strain involved inserting a URA3-KAN marker between the *SET1* promoter and the *SET1* open reading frame (ORF) and then replacing this marker with a sequence encoding the PTH tag. The URA3-KAN marker was amplified from pGSKU (54) by using oligonucleotides oCA164 and oCA165. The PTH tag was amplified on a plasmid expressing N-PTH-NPL3 (pRS415-PTH) by using oligonucleotides oCA167 and oCA168. The PTH-Set1ΔRRM2 strain was obtained from PTH-Set1. First, a URA3-KAN marker was amplified from pGSKU by using oligonucleotides oCA151 and oCA152 and integrated into RRM2 in the *SET1* ORF. The URA3-KAN marker was removed by using oligonucleotides as described previously (54). The oCA175 and oCA176 oligonucleotides are homologous to sequences upstream and downstream of RRM2, and their insertion resulted in a deletion from positions 243 to 482 of the *SET1* ORF and residues 415 to 494 of the Set1 protein. The HTP tag with a URA3 marker was amplified from pBS1539-HTP (55) and integrated to obtain the Set1-HTP and SET2-HTP strains. The Set1ΔRRM2 strain was obtained as described above for the PTH-Set1ΔRRM2 strain but starting from BY4741 instead of PTH-Set1. The *SET1* ORF was deleted by using a URA3 marker (Δset1:URA:pURA). The *URA3* coding sequence and promoter were inserted in the antisense direction relative to the *SET1* gene. In the W303 background, the *SET2* ORF was deleted by using a KanMX cassette (Δset2:KanMX).

### Immunoblotting.

For this study, we used the following antibodies: anti-H3 (catalog number Ab1791; Abcam), anti-H3K4me3 (catalog number 05-745; Upstate), anti-H3K4me2 (C64G9, catalog number 9725T; Cell Signaling Technology), anti-H3K4me1 (D1A9, catalog number 5626T; Cell Signaling Technology), anti-Set1 (catalog number yE-13; Santa Cruz Biotechnology), anti-Pgk1 (catalog number A-6457; Invitrogen), anti-H3K36me3 (catalog number Ab9050; Abcam), anti-H3K36me2 (catalog number Ab9049; Abcam), anti-H3K36me1 (catalog number Ab9048; Abcam), anti-goat (catalog number A-21446; Invitrogen), anti-mouse (catalog number A-21036; Invitrogen), and anti-rabbit (from Invitrogen [A-31537] or Abcam [Ab6721] for H3K36me blots) antibodies.

Cell extracts were prepared by using actively growing cells washed with water. Cells were lysed by vortexing with zirconia beads in TN150 buffer (50 mM Tris-HCl [pH 7.8], 150 mM NaCl, 0.1% NP-40, 5 mM β-mercaptoethanol, complete EDTA-free protease inhibitor cocktail [Roche]). The lysate was cleared by centrifugation. The protein concentration in the soluble extract was quantified by using a Bradford assay (56). The extract was denatured in NuPAGE sample buffer (Invitrogen) by incubation at 70°C for 10 min. A total of 15 to 50 μg of protein was resolved on 3 to 8% Tris-acetate NuPAGE gels (Invitrogen), 4 to 12% Bis-Tris NuPAGE gels (Invitrogen), or 15% SDS-polyacrylamide gels for Set1, Pgk1, and H3, respectively. Proteins were transferred onto nitrocellulose membranes, probed with the indicated antibodies, and imaged by using the Licor Odyssey system.

### *In vivo* RNA cross-linking.

Actively growing cells in synthetic dextrose (SD) medium with 2% glucose lacking tryptophan were UV cross-linked at 254 nm and processed essentially as described previously (55, 57).

Tagged proteins were recovered from total lysates by incubation with IgG-Sepharose for 2 h for Set2 or overnight for Set1 and eluted by TEV cleavage. The eluates were subjected to partial RNase degradation and denatured by the addition of 6 M guanidinium-HCl, and RNA-protein complexes were bound to nickel columns. The RNAs were labeled by using [γ-^32^P]ATP, and linkers were added to both ends, on the nickel column. The complexes were eluted with imidazole and resolved on 4 to 12% Bis-Tris or 3 to 8% Tris-acetate NuPAGE gels (Invitrogen) for Set2 and Set1, respectively, transferred onto nitrocellulose membranes, and detected by autoradiography. Bands corresponding to the size of the protein of interest were excised and incubated with proteinase K to release the bound RNAs. Phenol-purified RNAs were reverse transcribed and PCR amplified. Libraries were resolved on agarose gels, and fragments with insert sizes of approximately 20 to 80 bp were excised from the gel and sequenced by using Illumina HiSeq 50-bp single-end reads (Edinburgh Genomics or Source Bioscience). The reagents used were described previously (58).

### CRAC data analysis.

The data sets were demultiplexed by using pyBarcodeFilter from pyCRAC (59). FLEXBAR (60) was used to remove the 3′ sequencing adapters, trim low-quality positions from the 3′ ends of reads, and remove reads without a high-quality score (parameters –u 3 –q 30 –m 17 –ao 3). In addition to the barcode, the 5′ linkers contain a random 3-nt sequence, allowing PCR duplicates to be removed by collapsing identical sequences. Reads were filtered to exclude low-complexity sequences (with more than 80% of one nucleotide) to avoid potential non-genome-encoded oligo(A) tails mapping to A-rich regions of the genome (37). Reads were mapped to the yeast genome (S. cerevisiae genome version EF4.74, from Ensembl) by using novoalign from Novocraft (parameters –s 1 –r Random). To remove PCR duplicates that were not collapsed during preprocessing due to sequencing errors or differential trimming at the 3′ end, any reads with the same random tag in their 5′ linker and with 5′ ends mapping to the same genomic coordinate were collapsed (37).

We used genome annotation from Ensembl (EF4.74), supplemented with noncoding sequences as previously described (37). The distribution of reads across transcript classes was determined by using pyReadCounters from pyCRAC. The relative abundance of spliced and unspliced reads was calculated as described previously (33). The coverage at each position along the genome was calculated and normalized to the library size (reads per million) (33), after the exclusion of reads mapping to RNAPI and RNAPIII transcripts (including novel transcripts described previously [53] or originating from the mitochondrial genome). Replicate data sets were averaged. The enrichment of Set1, Set2, or phosphorylated RNAPII relative to total RNAPII was calculated as log_2_(protein coverage + 5/total Rpo21-HTP coverage + 5), where the pseudocount of 5 avoids numerical instabilities (33). Coverage around genomic features (metagene analyses and two-dimensional [2D] heat maps) was plotted as described previously (33). To compare Set1 or Set2 coverage to RNAPII coverage around genomic features, a subset of features with highly reproducible coverage within Set1 or Set2 replicate data sets (features for which the ratio of the standard deviation to the mean was below 0.5) and that were confidently bound (reads per kilobase per million [RPKM] over 30) were selected (4,851, 2,867, 4,306, and 4,199 features for PTH-Set1, PTH-Set1ΔRRM2, Set1-HTP, and Set2-HTP, respectively).

RNAPII CRAC data sets (33) (NCBI Gene Expression Omnibus [GEO] accession number GSE69676) were reprocessed with the pipeline described above.

### RT-qPCR.

RNA was isolated as described previously (61). The quantity and purity of RNA were analyzed by using a NanoDrop 1000 instrument. Two micrograms of total RNA was treated with RQ1 RNase-free DNase (Promega), and the reaction was stopped by phenol-chloroform extraction. Single-stranded cDNA was generated by using gene random primers (Thermo Scientific) and murine leukemia virus (MuLV) reverse transcriptase (Thermo Scientific). The expression level of individual transcripts was determined by quantitative PCR using SYBR Premix Ex *Taq* II Tli RNase H Plus (Clontech) for detection and by using oligonucleotides listed in Table S3 in the supplemental material. Relative levels were determined by normalization to the *ACT1* mRNA level in each sample. By using Prism (GraphPad Software, Inc.) and assuming normality, analysis of variance (ANOVA), followed by Dunnett's test, was performed to determine whether the relative expression level measured in each strain was significantly different from that measured in the wild-type BY4741 strain.

### RNA-seq.

Wild-type W303 and otherwise isogenic Δ*set2* cells (see Table S2 in the supplemental material) were grown in yeast extract, peptone, glucose, adenine sulfate (YPDA) to an optical density at 660 nm (OD_660_) of 0.6. Independent samples of total RNA were prepared from three WT and three Δ*set2* colonies by hot-phenol extraction. RNA was further subjected to DNase I treatment (catalog number E1009-A; Zymo Research) and Ribo-zero treatment (catalog number RZY1324; Illumina) according to the manufacturer's instructions. The quantity and purity of RNA were measured by using an Agilent high-sensitivity RNA screen tape system (catalog number 5067-5579; Agilent Technologies) and Qubit (Molecular Probes, Invitrogen). Libraries were prepared for sequencing from 200 ng of rRNA-depleted total RNA by using the NEXTflex RNA-seq kit (catalog number 5129-02; Bioo Scientific) according to the manufacturer's instructions. Samples were barcoded and combined at uniform molarity to create a single pool, which was sequenced in a single-end 76-bp run on an Illumina NextSeq machine. Multiplexed reads were split based on their NEXTflex barcodes, and 3′ adapter sequences were trimmed by using Illumina Basespace software. Trimmed reads were mapped to the sacCer3 genome by using tophat (62) with parameters –segment-length 38 –no-coverage-search –max-multihits 20 –report-secondary-alignments –read-mismatches 2. Mapped reads were filtered to remove reads mapping to more than one unique genomic locus (multihits) by keeping only reads with flag NH:i:1 in the output bam file from tophat. Reads were further filtered to remove reads with a mapping quality of <20 by using samtools (63).

Downstream analyses were conducted by using the statistical programming language R (R Development Core Team, 2008) and bioconductor packages. Transcriptome annotation was taken from Ensembl (EF4.74), supplemented with noncoding sequences as previously described (37). Read counts within transcriptional units were generated by using summarizeOverlaps() from the GenomicAlignments package (64) with parameters mode = “Union,” singleEnd = TRUE, inter.feature = TRUE, ignore.strand = TRUE, and fragments = FALSE. Differential expression analysis of Δ*set2* samples against WT samples was performed by using DESeq2 (65). Genes showing significant changes in expression levels in Δ*set2* samples were identified based on a fold change of >1.5 (up or down) and an adjusted *P* value (66) of <0.05.

### Set1 ChIP-qPCR.

The tagged strains PTH-Set1 and PTH-Set1ΔRRM2 and the untagged BY4741 strain were analyzed by ChIP. Actively growing cells in complete minimal medium at an OD of 0.5 were fixed for 15 min with 1% formaldehyde. The cross-linking reaction was quenched by the addition of 150 mM glycine. Cells were washed in cold phosphate-buffered saline (PBS), frozen in liquid nitrogen, and stored at −80°C. Cell pellets were disrupted in lysis buffer (50 mM HEPES-KOH [pH 7.5], 140 mM NaCl, 1 mM EDTA, 1% Triton X-100, 0.1% Na-deoxycholate, 0.1% SDS, and EDTA-free complete protease inhibitors from Roche Applied Science) by using a minibeadbeater. Unless stated otherwise, subsequent steps were performed at 4°C. The soluble lysate was discarded after centrifugation, and insoluble chromatin was resuspended in lysis buffer. Chromatin was sheared by 20 cycles of sonication with 30 s on and 30 s off using a Bioruptor 300 instrument (Diagenode) at high power, leading to fragments of 0.1 to 1 kb. The solubilized chromatin was separated from insoluble debris by centrifugation. A total of 1.5 mg of chromatin was used for immunoprecipitation (IP), and 37.5 μg was used as input samples. ChIP was performed by incubating the lysate with rabbit IgG (catalog number 15006; Sigma) coupled with Dynabeads M270 epoxy (Invitrogen) for 2 h. Beads were washed for 15 min with each of the following buffers: lysis buffer, 0.5 M lysis buffer (same as lysis buffer but with 500 mM NaCl), wash buffer (10 mM Tris-HCl [pH 8], 0.25 M LiCl, 0.5% NP-40, 0.5% Na-deoxycholate, 1 mM EDTA), and Tris-EDTA (TE) (10 mM Tris-HCl [pH 8], 1 mM EDTA). Beads were resuspended in elution buffer (50 mM Tris-HCl [pH 8], 10 mM EDTA, 1% SDS), and cross-linking was reverted by overnight incubation at 65°C. Samples were treated with 0.25 mg/ml of proteinase K (Roche) at 55°C for 4 h and with 0.2 mg/ml of RNase A (Thermo Scientific) at 37°C for 2 h. DNA was purified by using the QIAquick kit (Qiagen), elution buffer was supplemented with 0.2 mg/ml of RNase A, and the eluted DNA was incubated at 37°C for 2 h.

Relative DNA amounts present in input samples and purified fractions were determined by qPCR using SYBR Premix Ex *Taq* II (Clontech). Primer pairs used for amplification are listed in Table S3 in the supplemental material. All samples were run at least in triplicate. The mean values and standard errors were derived from three biological replicates. Using Prism (GraphPad Software, Inc.) and assuming normality, Student's *t* tests were performed to determine the *P* value for the differences in percentages of input DNA obtained for PTH-Set1 and PTH-Set1ΔRRM2, for each primer pair. Results, including those for the BY4741 negative control, are included in Table S4 in the supplemental material.

### H3 ChIP-qPCR.

The wild-type and Set1ΔRRM2 strains were grown and cross-linked as described above for Set1 ChIP. Unless stated otherwise, subsequent steps were performed at 4°C. Cells were disrupted in lysis buffer (20 mM Tris-HCl [pH 8], 150 mM NaCl, 2 mM EDTA, 1% Triton X-100, 0.1% SDS, EDTA-free complete protease inhibitors from Roche Applied Science, and 0.5 mM phenylmethylsulfonyl fluoride [PMSF]) by using a FastPrep instrument (MP Biomedicals). Chromatin was sheared by sonication with 5 s on and 5 s off at 95% amplitude for 3 h by using a Q800R2 sonicator (Qsonica). IP buffer (167 mM Tris-HCl [pH 8], 167 mM NaCl, 1.2 mM EDTA, 1.1% Triton X-100, 0.01% SDS, 0.5 mM PMSF, and complete protease inhibitors from Roche) was added to the solubilized chromatin and incubated for 15 min. Fifty microliters of chromatin was used as input DNA. ChIP was performed by overnight incubation of 1 ml of chromatin with antibodies (from Abcam) against H3 (catalog number ab1791), H3K4me1 (catalog number ab8895), H3K4me2 (catalog number ab7766), H3K4me3 (catalog number ab8580), or green fluorescent protein (GFP) as a negative control (catalog number ab290), followed by 2 h of incubation with Dynabeads-protein A (Invitrogen). Beads were washed with TSE-150 buffer (20 mM Tris-HCl [pH 8], 150 mM NaCl, 2 mM EDTA, 1% Triton X-100, 0.1% SDS), TSE-500 (same as TSE-150 buffer but with 500 mM NaCl), wash buffer (10 mM Tris-HCl [pH 8], 0.25 M LiCl, 1% NP-40, 1% Na-deoxycholate, 1 mM EDTA), and TE (10 mM Tris-HCl [pH 8], 1 mM EDTA). DNA was eluted at 65°C in elution buffer (100 mM NaHCO_3_, 1% SDS), and cross-linking was reverted, after the addition of 500 mM NaCl, by overnight incubation at 65°C. Samples were treated with 0.5 mg/ml of RNase A at 37°C for 2 h. DNA was purified by using the ChIP DNA Clean & Concentrator kit (Zymo Research).

Relative DNA amounts were determined by qPCR using primer pairs listed in Table S3 in the supplemental material. The mean values and standard errors were derived from three biological replicates. By using Prism (GraphPad Software, Inc.) and assuming normality, Student's *t* tests were performed to determine the *P* value for the differences in relative enrichment compared to total H3 obtained for the wild type and Set1ΔRRM2, for each primer pair. Results, including those for the negative controls, are included in Table S5 in the supplemental material.

### Accession number(s).

CRAC and RNA-seq sequences generated during this work have been deposited in the GEO database (http://www.ncbi.nlm.nih.gov/geo/) under accession numbers GSE87919 and GSE89238.

## Supplementary Material

Supplemental material
