# Supplementary material for: RNA Binding by Histone Methyltransferases Set1 and Set2
Source: Mol Cell Biol. 2017 Jun 29;37(14):e00165-17. doi: 10.1128/MCB.00165-17 (PMC5492175; doi:10.1128/MCB.00165-17)
Supplement: Supplemental material [file supp_37_14_e00165-17__index.html]

Supplemental material 

# RNA Binding by Histone Methyltransferases Set1 and Set2

## Supplemental material

- Supplemental file 1 -

  Table S1 (Number of reads mapping to RNAPII transcripts), S2 (Yeast strains), S3 (Oligonucleotides), S4 (Set1 ChIP-qPCR expressed as percentage of input DNA), and S5 (Methylated H3K4 ChIP in wild-type and Set1ΔRRM2 strains) and Fig. S1 (Protein level and cross-linking efficiency in different strains), S2 (Set1 and Set2 relative enrichment and mRNA stability), S3 (Set1, Set2, and RNAPII distribution across transcripts), S4 (Set1 binding to *SET1* mRNA and transcript abundance), S5 (Enrichment for Set1 or Set2 relative to RNAPII on transcripts), and S6 (Global methylated H3K4 levels in Set1 and Set1ΔRRM2)

  PDF, 5.3M
