# Supplementary material for: RNA Binding by Histone Methyltransferases Set1 and Set2
Source: Mol Cell Biol. 2017 Jun 29;37(14):e00165-17. doi: 10.1128/MCB.00165-17 (PMC5492175; doi:10.1128/MCB.00165-17)

RNA binding by the histone methyltransferases Set1 and Set2
Sayou et al., 2017

SUPPLEMENTARY INFORMATION

Supplemental tables

Table S1. Number of reads mapping to RNAPII transcripts (any read mapping to the nuclear genome, outside of RNAPI and RNAPII transcribed genes), where PCR duplicates have been removed, in the CRAC datasets used in this study.

PTH-Set1	PTH-Set1_1	409896
	PTH-Set1_2	207591
	PTH-Set1_3	105599
	PTH-Set1_4	138665
PTH-Set1ΔRRM2	PTH-Set1dRRM2_1	337413
	PTH-Set1dRRM2_2	68136
	PTH-Set1dRRM2_3	17417
Set1-HTP	Set1-HTP_1	406822
	Set1-HTP_2	648139
	Set1-HTP_3	75245
	Set1-HTP_4	71919
Set2-HTP	Set2-HTP_1	182583
	Set2-HTP_2	136682
	Set2-HTP_3	209546
BY4741	BY4741_1	882
	BY4741_2	1162

Table S2. Yeast strains

**Name**	**ID**	**Genotype**	**Reference**
BY4741	BY4741	*MAT*a; *his3Δ1*; *leu2Δ0*; *met15Δ0*; *ura3Δ0*	(67)
PTH-Set1	yCA14	*MAT*a; *his3Δ1*; *leu2Δ0*; *met15Δ0*; *ura3Δ0; PTH-SET1*	This study
PTH-Set1ΔRRM2	yCA18	*MAT*a; *his3Δ1*; *leu2Δ0*; *met15Δ0*; *ura3Δ0; PTH-*	This study

		SET1ΔRRM2 (deletion nucleotides 243-482)	
Set1-HTP	D1202	*MAT*a; *his3Δ1*; *leu2Δ0*; *met15Δ0*; *ura3Δ0; SET1-HTP:URA3*	This study
Set1ΔRRM2	yCA33	*MAT*a; *his3Δ1*; *leu2Δ0*; *met15Δ0*; *ura3Δ0; SET1ΔRRM2 (deletion nucleotides 243-482)*	This study
Δset1:URA:pURA	yCA28	*MAT*a; *his3Δ1*; *leu2Δ0*; *met15Δ0*; *ura3Δ0; Δset1:URA3:pURA3*	This study
Set2-HTP	Set2-HTP	*MAT*a; *his3Δ1*; *leu2Δ0*; *met15Δ0*; *ura3Δ0; SET2-HTP:URA3*	This study
Rpo21-HTP	Rpo21-HTP	*MAT*a; *his3Δ1*; *leu2Δ0*; *met15Δ0*; *ura3Δ0; RPO21-HTP:URA3*	(33)
W303	W303	*MATa; his3-11,-15; leu2-3,-112; trp1-1; ura3-1; ade2-1; can1-100*	R. Rothstein
Δset2	Δset2	*MATa; his3-11,-15; leu2-3,-112; trp1-1; ura3-1; ade2-1; can1-100; Δset2:HIS3*	This study

Table S3. Oligonucleotides

**Strain construction**			
**Name**	**Sequence**	**Description**	**Reference**
oCA164	TGTCTGTATGAACCAGAAGACGCGTG TGCTCTTCTATAGTAATTTGACATTTC GTACGCTGCAGGTCGAC	Amplify a URA-KAN marker from pGSKU and integrate in between pSET1 and SET1 ORF	This study
oCA165	TAACATTCCTTATTTGTTGAATCTTTAT AAGAGGTCTCTGCGTTTAGAGATAGG GATAACAGGGTAATCCGCGCGTTGGC CGATTCAT		
oCA167	TAACATTCCTTATTTGTTGAATCTTTAT AAGAGGTCTCTGCGTTTAGAGAGAGG AGAAATTAACCATGAACA	Amplify the PTH tag and replace the URA-KAN marker upstream SET1 ORF	This study
oCA168	GGTTGTCTGTATGAACCAGAAGACGC GTGTGCTCTTCTATAGTAATTTGATCC GGCATGGTGGTGATGGTG		
oCA151	GCACATCTGTATCCCCTTAACTTGTAT	Amplify a URA-KAN	This study

	TTAAAGTCCTCAACGCAGAACCTTTCG TACGCTGCAGGTCGAC	marker from pGSKU and integrate in the RRM2 region in SET1 ORF	
oCA152	TTAATAACAGACCTGTTTTACATGTCT CCAAAATATTTGTTGCCAAACATTAGG GATAACAGGGTAATCCGCGCGTTGGC CGATTCAT		
oCA175	CTCATTCGTCCGGAAGTGAAAAAAGA ATTCCATATGATCTCTTGGGGGTACTC ATTCTCC	Replace the URA-KAN marker in the RRM2 region in SET1 ORF and delete RRM2	This study
oCA176	GAGCTAGATTTATTGAATCTAGTTCTG TTTTGGAACCTAGGGAGAATGAGTAC CCCCAAG		
SET1-HisFw	GGAAAGACTTCCTTGTTTATGTGGAGC ACCTAATTGTAAAGGTTTCTTGAACGA GCACCATCACCATCACC	Amplify the HTP tag and URA marker and integrate udownstream SET1 ORF	This study
SET1-TAP Rev	GGAAGGCAACGATATGTTAAATCAGG AAGCTCCAAACAAATCAATGTATCATC GTACGACTCACTATAGGG		
oCA260	TATCATCAGCATCAACAAGGATGTCTT CTCCTCCACCTTCAACATCATCAGAGC ACCATCACCATCACC	Amplify the HTP tag and URA marker and integrate udownstream SET2 ORF	This study
oCA261	CTTTGGGACAGAAAACGTGAAACAAG CCCCAAATATGCATGTCTGGTTAAACG ACTCACTATAGGG		
oCA201	TAACATTCCTTATTTGTTGAATCTTTAT AAGAGGTCTCTGCGTTTAGAGACTCC TTACGCATCTGTGCG	Delete SET1 ORF with URA3:pURA3	This study
oCA202	AGCAACGATATGTTAAATCAGGAAGCT CCAAACAAATCAATGTATCATCGGGCA TCAGAGCAGATTGTACTGA		
**RT-qPCR**			
**Name**	**Sequence**	**Target gene(s) or region**	**Reference**
ACT1-F	GAAATGCAAACCGCTGCTCA	*ACT1*	This study
ACT1-R	TACCGGCAGATTCCAAACCC		

oCA246	GATGGTACAAGGCGACGCTA	*SET1*	This study
oCA247	ATGCCCCTCCGACTACTGAT		
oCA282	ACCAGGAAACGACGGTTTGT	*ORC6*	This study
oCA283	CGCTTCTGCTTTCTTGCACA		
oCA225	TGAGAGGTCTATCTGGCGAA	All TYA encoding genes	This study
oCA226	CCGAGCTATAACTTTGGGTTTGG		
Ty-SetC-F	TCACTACACCACGTCGTTCC	*YLR035C-A, YHR214C-B, YER160C*	(18)
Ty-SetC-R	GGAGTGGAAGATCAGCGATAA		
p17-F	GGAAAGCGGGAAGGAATAAG	IGS1, region of the rDNA repeat between *RDN37* and *RDN5*	(68)
p17-R	CGATTCAGAAAAATTCGCACT		
oCA235	TGGGGTGGTATAGTCCGCAT	IGS2, region of the rDNA repeat between *RDN5* and *RDN37*	This study
oCA236	TCGGTGACGGAAATACGCTT		
**ChIP-qPCR**			
**Name**	**Sequence**	**Target gene(s) or region**	**Reference**
PMA1_1up	GGTACCGCTTATGCTCCCCTCCAT	*PMA1*	(32)
PMA1_1low	ATTTTTTTTCTTTCTTTTGAATGTGTG		
PMA1_2up	AAGTCGTCCCAGGTGATATTTTGCA		
PMA1_2low	AACGAAAGTGTTGTCACCGGTAGC		
PMA1_3up	CAGAGTTGTTGAAATCTTGC		
PMA1_3low	TGTCTGGAGGTCTTCAAAGC		
PMA1_4up	TCATCGCTACCATGTTTACC		
PMA1_4low	CTTCATTGGCTTACCGTTCATC		
oCA262	GTTGTCGTTATCGGTCATGTCG	*TEF1, TEF2*	This study
oCA263	CTTGTCCAAAACCCAAGCGT		
oCA268	CGACCCATTCATCACCAACG	*TDH3*	This study
oCA269	ACCCCATGGCAAGTTAGCTG		
oCA272	TCTGCAACTCCCGTGTCATC	*ILV5*	This study
oCA273	GGCCAGTCAGCTCTTTCGTA		
oCA279	CAACAACGGTGACAGCTTCG		

Table S4. Set1 ChIP-qPCR expressed as percentage of input DNA from biological triplicates. Protein A beads were used for ChIP in BY4741 (untagged strain), PTH-Set1 and PTH-Set1ΔRRM2.

Primer pairs	Mean			Standard deviation		
	PTH-Set1	PTH-Set1ΔRRM2	BY4741	PTH-Set1	PTH-Set1ΔRRM2	BY4741
**PMA1-1**	0,0403	0,0282	0,0009	0,0031	0,0043	0,0003
**PMA1-2**	0,0672	0,0456	0,0017	0,0045	0,0022	0,0004
**PMA1-3**	0,0301	0,0329	0,0024	0,0028	0,0012	0,0008
**PMA1-4**	0,0254	0,0289	0,0020	0,0010	0,0017	0,0009
**TEF1**	0,1009	0,0766	0,0013	0,0081	0,0159	0,0003
**TDH3**	0,1436	0,0967	0,0014	0,0304	0,0204	0,0002
**ILV5**	0,1070	0,0784	0,0015	0,0170	0,0158	0,0003

Table S5. Methylated H3K4 ChIP in the wild-type (expressing endogenous Set1) and Set1ΔRRM2 strains from biological triplicates. Antibodies against H3K4me3, H3K4me2 and H3K4me1, or GFP (negative control) have been used for ChIP. The signal is normalized the total H3 signal.

Primer pairs	Antibody	Mean		Standard deviation	
		Set1	Set1ΔRMM2	Set1	Set1ΔRMM2
**PMA1-1**	**H3K4me1**	0,408	0,395	0,095	0,094
	**H3K4me2**	3,024	4,526	0,478	0,762
	**H3K4me3**	3,087	1,888	0,678	0,307
	**GFP**	5,869E-05	1,040E-04	1,562E-05	1,257E-04
**PMA1-2**	**H3K4me1**	0,428	0,437	0,120	0,095
	**H3K4me2**	0,983	1,230	0,212	0,316
	**H3K4me3**	0,489	0,404	0,084	0,099
	**GFP**	4,298E-05	9,150E-05	3,671E-05	1,026E-04
**PMA1-3**	**H3K4me1**	0,202	0,210	0,052	0,038
	**H3K4me2**	0,312	0,441	0,063	0,089
	**H3K4me3**	0,118	0,116	0,013	0,032

	**GFP**	8,729E-05	1,252E-04	8,586E-05	4,215E-05
**PMA1-4**	**H3K4me1**	0,208	0,218	0,052	0,031
	**H3K4me2**	0,311	0,389	0,072	0,030
	**H3K4me3**	0,122	0,129	0,010	0,033
	**GFP**	8,352E-05	1,065E-04	7,269E-05	1,115E-04
**TDH3**	**H3K4me1**	0,188	0,193	0,047	0,029
	**H3K4me2**	2,105	3,779	0,726	0,768
	**H3K4me3**	5,135	3,468	0,335	0,645
	**GFP**	2,862E-05	1,771E-05	1,534E-05	1,124E-05
**TEF1**	**H3K4me1**	0,084	0,087	0,037	0,016
	**H3K4me2**	1,196	2,388	0,376	0,232
	**H3K4me3**	8,341	5,773	1,398	0,738
	**GFP**	2,636E-05	1,512E-05	2,852E-06	1,552E-06
**ILV5**	**H3K4me1**	0,113	0,133	0,040	0,016
	**H3K4me2**	1,165	3,084	0,376	0,551
	**H3K4me3**	4,755	3,074	0,131	0,960
	**GFP**	1,437E-04	6,050E-05	1,728E-04	5,766E-06

Supplemental Figures and legends

Figure S1. Protein level and crosslinking efficiency in the different strains.

A. Protein abundance and H3K4me3 levels in the Set1 strains used in this study, detected by western-blot. Yeast cells were grown in rich medium. * indicates a non-specific band detected with the anti-Set1 antibodies.

B. H3K36 mono-, di- and tri-methylation levels in the Set2-HTP, the isogenic Δset2 and the untagged (BY4741) strains, detected by western-blot. Yeast cells were grown in minimal media lacking tryptophan. Bellow each H3K36me blot is the H3 blot obtained from the same membrane.

C. Growth curves of the different strains used in this study. Cells were grown in minimal media lacking tryptophan.

D. SDS-PAGE and autoradiography of the 5' [^{32}P] labeled, crosslinked RNAs after purification of the Set1-HTP protein, or after mock purification from the untagged strain (BY4741).

E. Number of reads recovered from a CRAC experiment where crosslinked and barcoded samples from PTH-Set1, PTH-Set1ΔRRM2 and from the untagged strain (BY4741) were mixed prior to SDS-PAGE separation and RT-PCR amplification.

Figure S2. Set1 and Set2 relative enrichment and mRNA stability.

A-D. PTH-Set1 (A), PTH-Set1ΔRRM2 (B), Set1-HTP (C), Set2-HTP (D) enrichment relative to RNAPII (Rpo21-HTP) across mRNAs is plotted against the mRNA half-lives (38).

Figure S3. Set1, Set2 and RNAPII distribution across transcripts.

A-D. Coverage, in reads per million of RNAPII transcripts, for selected individual protein coding genes: *PMA1* (A), *RPS13* (B), *TEF1* (C), *RPL3* (D). Transcription units are represented under the plots with thicker boxes correspond to coding sequences.

E-F. Distribution of PTH-Set1 (E) and Set2-HTP (F) enrichment relative to total RNAPII, across individual mRNAs. Transcripts are aligned to the TSS and pA site in the left and right panels, respectively. Distances are indicated in nucleotides. Blue color indicates depletion, and red color indicates enrichment relative to total RNAPII.

G. Metagene analysis of PTH-Set1, Set2-HTP, RNAPII-S2P and RNAPII-S5P enrichment relative to total RNAPII across mRNAs (left), SUTs (middle) and CUTs (right) with similar expression levels, based on their total RNAPII CRAC signal over the first 300 nt (33). Each subset contains 211 to 213 transcripts.

Figure S4. Set1 binding to *SET1* mRNA and retrotransposons, and transcripts abundance in the different strains.

A. Set1-HTP coverage, in reads per million of RNAPII transcripts, at the *SET1* locus. The transcription unit is represented under the plot with the thicker box corresponding to the coding sequence. The corresponding plots for PTH-Set1 and RNAPII are shown in Figure 3C.

B. PTH-Set1 and RNAPII coverage, in reads per million of RNAPII transcripts, at a retrotransposon locus. *YML045W-A* and *YML045W* are coding for TYA and TYA-TYB, respectively. The LTRs are shadowed on the plots.

C-E. Transcript level measured by RT-qPCR. * indicates a relative expression different from that of BY4741 with a p-value bellow 0.01, calculated with a Dunnett's test. *SET1* mRNA (C), IGS transcripts (D) and retrotransposons (E) have been assessed.

Figure S5. Enrichment for Set1 or Set2 relative to RNAPII on transcripts.

A. PTH-Set1dRRM2 coverage over genomic features is plotted against PTH-Set1 coverage. The different transcript classes have been plotted separately on different panels. Selected classes of transcripts have been highlighted, as indicated below the plots.

B. PTH-Set1dRRM2 coverage over genomic features is plotted against RNAPII coverage (Rpo21-HTP). Note that the fill color of the points represents the enrichment for PTH-Set1 relative to RNAPII.

C. PTH-Set1 (top), Set2-HTP (bottom) coverage over genomic features is plotted against RNAPII coverage. The fill color of the points represents the enrichment for Set1 or Set2 relative to RNAPII (as in Figure 3A-B).

D. PTH-Set1 (left) or Set2-HTP (right) coverage over mRNAs is plotted against RNAPII coverage (Rpo21-HTP). The fill color of the points represents the enrichment for Set1 or Set2 relative to RNAPII. mRNAs found as differentially expressed in *set1*Δ (18) or *set2*Δ (this study) compared to wild-type in RNA-seq analysis are highlighted in green.

Figure S6. Global methylated H3K4 levels in Set1 and Set1ΔRRM2.

A. H3K4me3, H3K4me2, H3K4me1, H3 (loading) and Pgk1 (loading) levels in the wild type (Set1), Set1ΔRRM2 and Δset1 stains, detected by western-blot. Yeast cells were grown in minimal medium, each line results from an independent clone.

B. Quantification of the western-blots shown in A, were the signals have been normalized to H3.

Supplemental references

67. Brachmann CB, Davies A, Cost GJ, Caputo E, Li J, Hieter P, Boeke JD. 1998. Designer deletion strains derived from Saccharomyces cerevisiae S288C: a useful set of strains and plasmids for PCR-mediated gene disruption and other applications. Yeast 14:115–132.

68. Huang J, Moazed D. 2003. Association of the RENT complex with nontranscribed and coding regions of rDNA and a regional requirement for the replication fork block protein Fob1 in rDNA silencing. Genes Dev 17:2162–2176. https://doi.org/10.1101/gad.1108403.

Supplemental Material
Sayou et al.
RNA binding by the histone methyltransferases Set1 and Set2
Figure S1

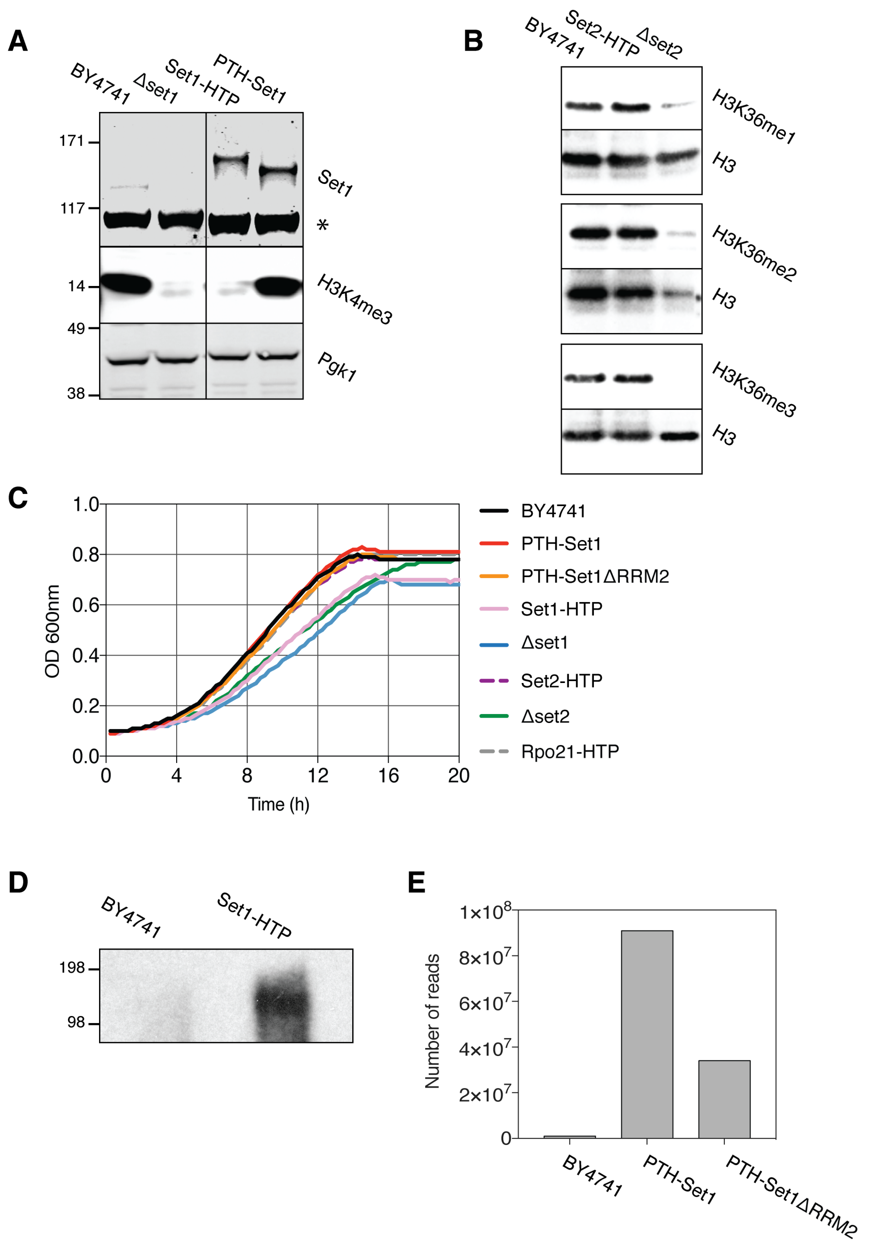

Supplemental Material
Sayou et al.
RNA binding by the histone methyltransferases Set1 and Set2
Figure S2

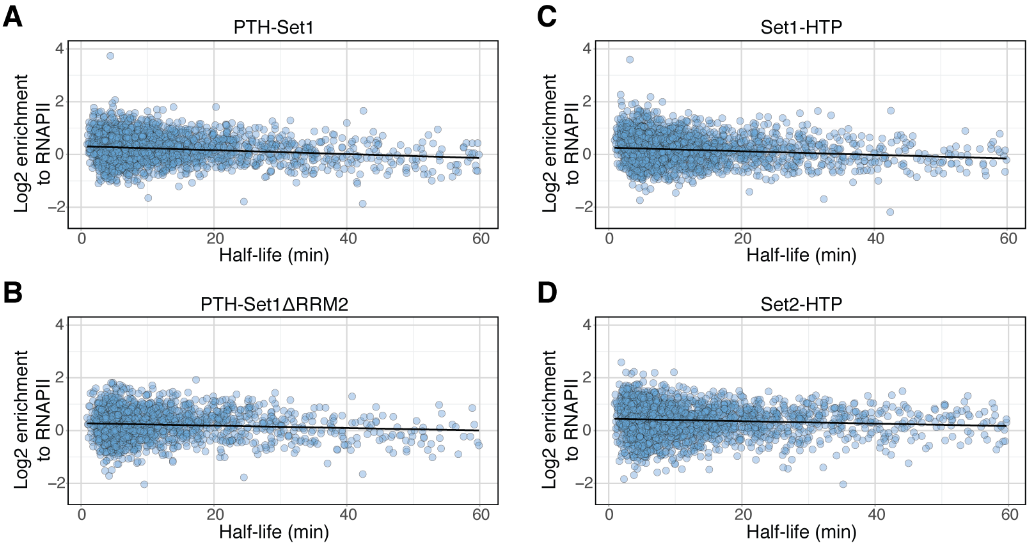

Supplemental Material
Sayou et al.
RNA binding by the histone methyltransferases Set1 and Set2
Figure S3

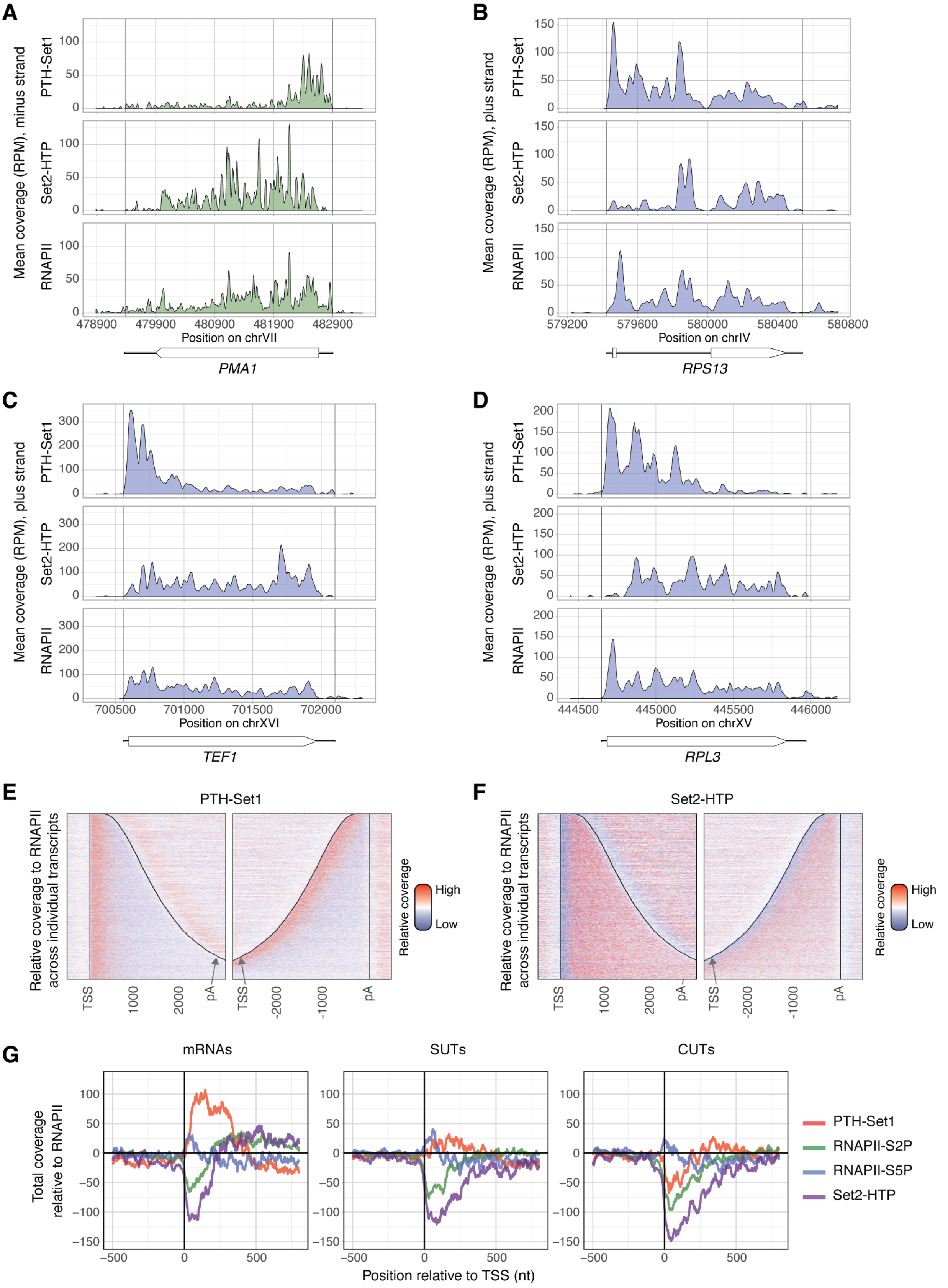

Supplemental Material
Sayou et al.
RNA binding by the histone methyltransferases Set1 and Set2
Figure S4

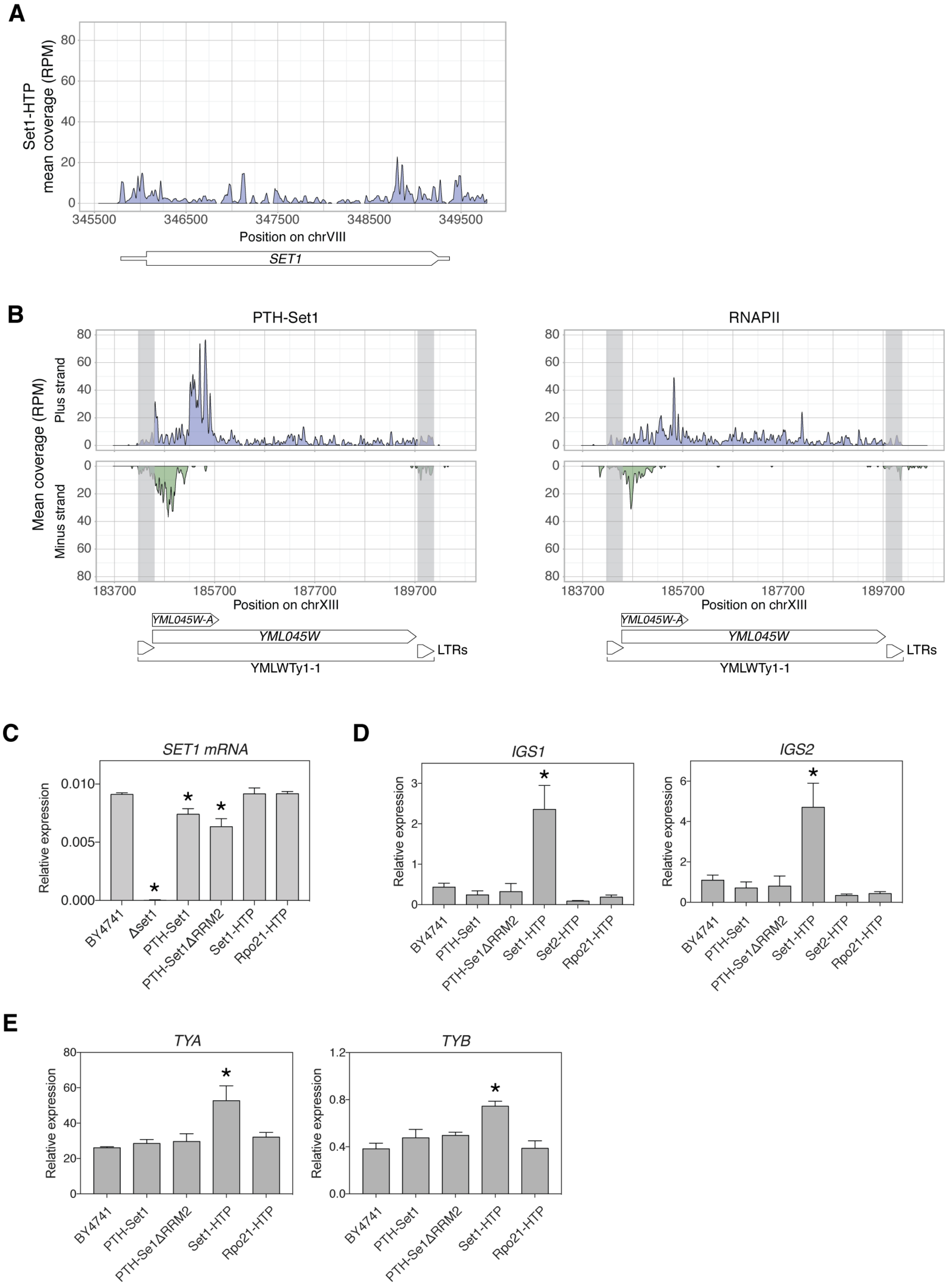

Supplemental Material
Sayou et al.
RNA binding by the histone methyltransferases Set1 and Set2
Figure S5

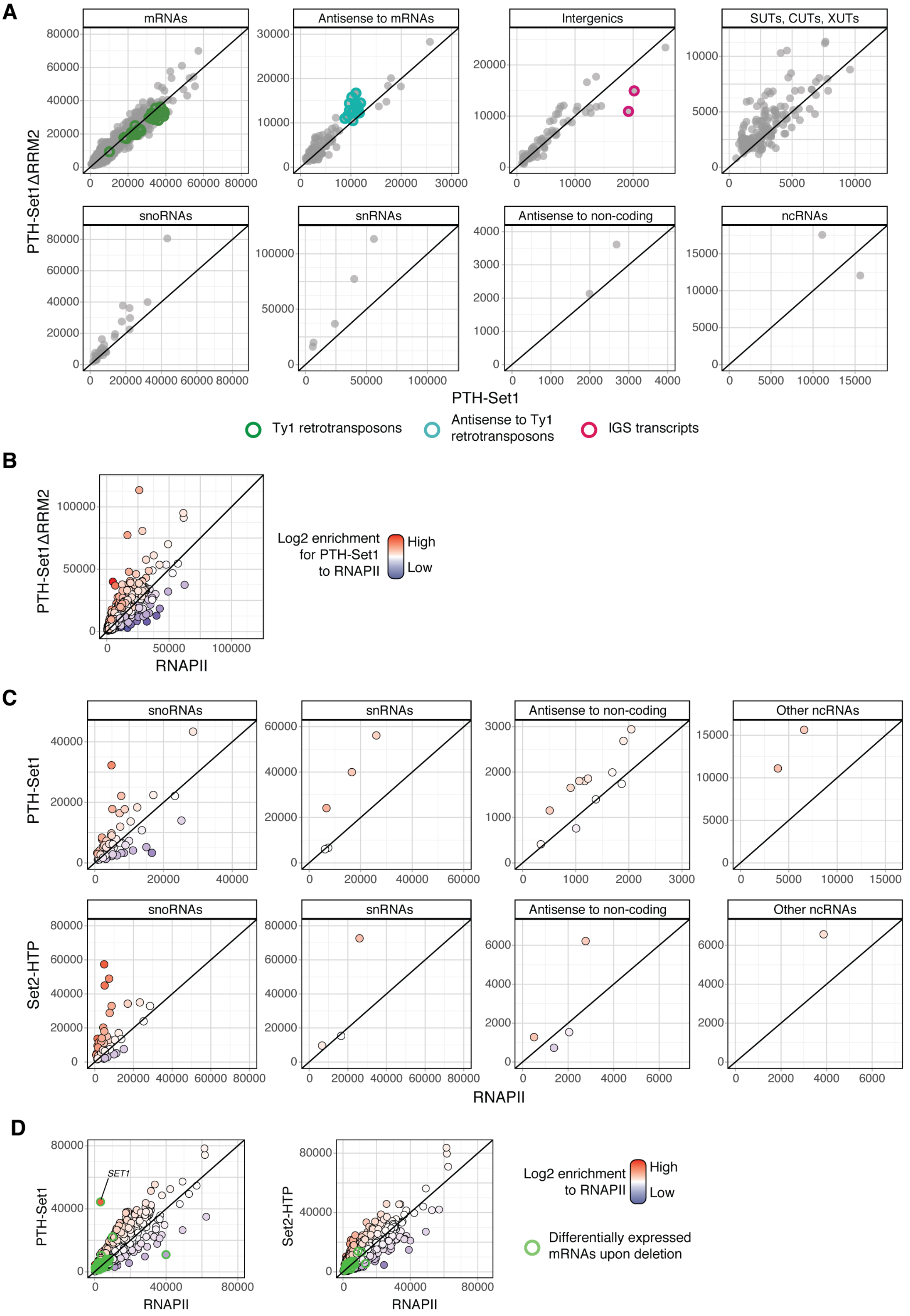

Supplemental Material
Sayou et al.
RNA binding by the histone methyltransferases Set1 and Set2
Figure S6

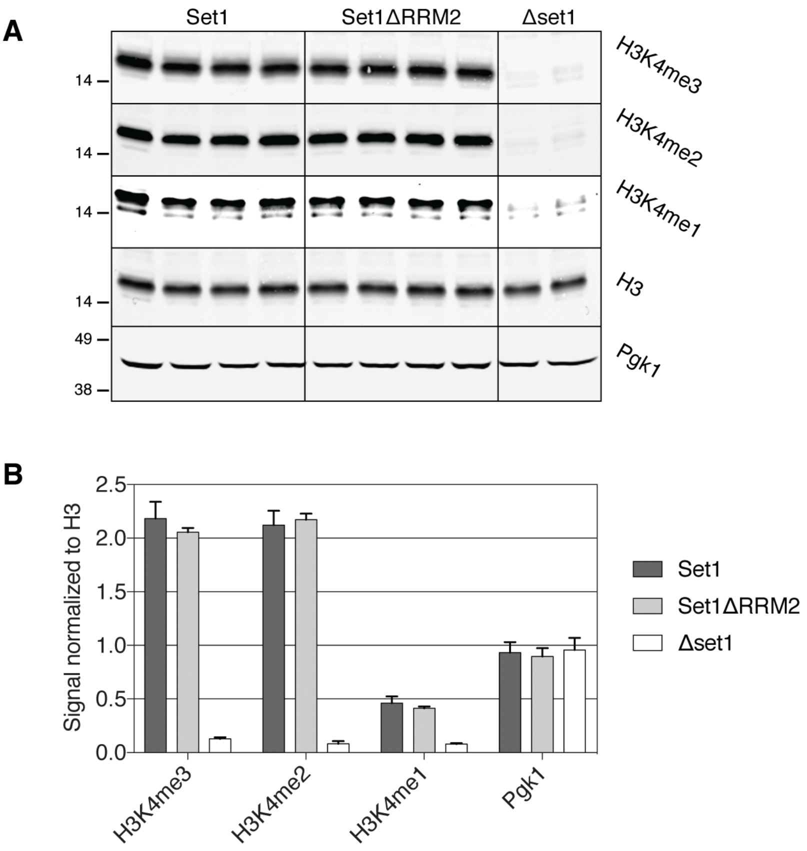

Supplement: Supplemental material [file MCB.00165-17_zmb999101530s1.pdf]
